# Hypolipidemic and Renal Functionality Potentials of the Hexane Extract Fractions of *Elephantopus Scaber* Linn

**Published:** 2010-09

**Authors:** P. Daisy, Cecilia Edel Priya

**Affiliations:** *P.G and Research Department of Biotechnology and Bioinformatics, HolyCross College, Trichy, India*

**Keywords:** diabetes, *Elephantopus scaber*, fractions,hypolipidemic and renal function

## Abstract

**Background:**

Abnormalities in lipid profile are one of the most common complications in diabetes mellitus. In STZ-induced diabetic rats, the rise in blood glucose is accompanied by a disturbance in lipid profile. Further still is the disturbance in the renal functions which includes abnormality in the serum urea,protein and creatinine levels The aim of the present study is to prove the hypolipidemic and the renal functionality effect of the root of *Elephantopus scaber* Linn.

**Procedure:**

Fractions obtained from crude extracts of hexane prepared from the root of *Elephantopus scaber* Linn. were administered to white albino rats at a dose of 0.15 g/Kg bwt for a period of 30 days to prove its hypolipidemic effect on a long term basis which is proved to be irreversible as the plant is reported to have regenerative property.

**Findings:**

The extract produced a significant (*p*<0.001) dose-dependent decrease in the levels of total cholesterol (TC), Triacylglycerol (TG), low-density lipoprotein-cholesterol (LDL cholesterol), with a significant increase in the level High-density lipoprotein-cholesterol (HDL) and reporting the restoration in renal functions back to near normal.

**Conclusion:**

The fractions of *Elephantopus scaber* Linn. hexane extract are confirmed to have a hypolipidemic potential and a re-establishment of renal functions.

## INTRODUCTION

In the Indian system of medicine, the medicinal attribution of this species has been for a long time. As per the traditional claims, the roots were used as an antipyretic, cardio tonic, and diuretic (14). Abnormalities in lipid profile are one of the most common complications in diabetes mellitus, which is found in about 40% of diabetics (17). Insulin deficiency or insulin resistance is associated with hypercholesterolemia and hypertriglyceridemia (23). Diabetes induction causes increase in the cholesterol, triglycerides, VLDL and LDL (22). Coronary artery disease, as a result of premature atherosclerosis, is a major cause of death both in type I and II diabetes *Elephantopus scaber* Linn. (Asteraceae) is a scaberescent aromatic herb distributed in the moist deciduous forests of the central Western Ghats. The plant is popularly known as Elephant’s foot (English), Gojivha (Sanskrit), Eddumalikechettu (Telugu) and Nayi nalige (Kannada). Genus Elephantopus of family Asteraceae is found in tropical countries and contains 32 species (27) diabetes (1). The aqueous extract of leaves is applied externally to treat eczema and ulcers (5). The whole plant is macerated and applied on the wound surface (25) to promote wound healing. The infusion and decoction of the whole plant, roots and leaves are used in folk medicine for the treatment of fever and to eliminate bladder stones (10). The plant extract is bitter, acrid, astringent, antipyretic, antidiabetic, diuretic and tonic. A decoction of the roots and leaves are given in dysuria, intermittent fevers, diarrhea and bronchitis and especially for haemorrhoids and various other diseases conditions (26). The plant extract also shows anticancer property (11). The pharmacological properties of the leaf extracts have been evaluated for diuretic (18), anti-inflammatory and hepatoprotective properties (11).

## MATERIALS AND METHODS

### Preparation of the fraction

The species were identified and authenticated at the Department of Botany, Holy Cross College and voucher specimen deposited. Root of *Elephantopus scaber* was washed well and shade dried. It was ground completely to form coarse powder 2 Kg Powder was soaked in 4 L of hexane Ethyl acetate and methanol and was extracted serially in Rotary evaporator under reduced pressure. The yield obtained was 10.6 W/W. The dry residue resulting was stored at 4°C. The hexane crude extract yielded 55 fractions with 100% hexane column and 5 fractions with hexane: chloroform (80:20) column. The pooling was done for the above fractions using the Rf value based on the spotting on a precoated silica gel 60 F254, 0.25 mm thick TLC plate (Merck). 6 fractions were obtained after pooling (5 fractions in 100%hexane column and 1 pooled fraction from hexane: chloroform column.

### Chemicals

Streptozotocin was purchased from Sigma-Aldrich, St.Louis USA. All other commercial reagents used were of commercial grade.

Wistar male albino rats weighing 100-150 g were obtained from King’s Institute for the study. The animals were fed on pellet diet (Sai Durga Foods and Feeds, Bangalore) and water ad libitum. Animals were maintained in the Animal house at an ambient temperature at 12 h dark and light cycle. The Animal ethical committee was approved (Reg. No 585/05/A/CPCSEA).

### Induction of Diabetes

A freshly prepared solution of streptozotocin in pH 4.5 dissolved in citrate buffer saline was intraperitonially administered in male Wistar rats weighing 100-150 g. They were made diabetic with a dose of 60 mg/Kg bwt. After 1 week the animals with blood sugar level of 300-350 were taken for the study.

9 groups of animals were taken for the study. Each containing 2 rats.

Group1: Diabetic rats (administered with 60 mg/Kg bwt of STZ);
Group2: Normal rats (not induced with STZ);
Group3: Fraction 1 (0.15 g/Kg bwt) daily using intragastric tube;
Group4: Fraction 2 (0.15 g/Kg bwt) daily using intragastric tube;
Group5: Fraction 3 (0.15 g/Kg bwt) daily using intragastric tube;
Group6: Fraction 4 (0.15 g/Kg bwt) daily using intragastric tube;
Group7: Fraction 5 (0.15 g/Kg bwt) daily using intragastric tube;
Group8: Fraction 6 (0.15 g/Kg bwt) daily using intragastric tube;
Group9: Insulin treated diabetic rat (3 I.U/kg bwt) daily using Insulin Syringe.

### Drug Administration

Twenty four hours after the last administration, the animals were anaesthetized with chloroform vapor and dissected. Whole blood was obtained by cardiac puncture from each rats and collected into sample bottles. The serum and tissue samples (liver) were kept at -20°C until assayed for Biochemical parameters.

Serum total cholesterol, triglyceride and High density Lipo-protein (HDL) were measured by enzymatic colorimetric method using Diagnostic kit-Beacon Diagnostics, Kabilpore, Navsari, India, for total cholesterol and HDL estimations. Diagnostic kit-Bio systems, Costa Brava, Spain for the estimation of triglycerides. The concentration of total cholesterol, estimation of triglycerides for was calculated by the formula:

Absorbance of sample/Absorbance of standard*200=mg/dl (Estimation of total cholesterol and triglycerides).

Absorbance of sample/Absorbance of standard*100*1.1=mg/dl (Estimation of serum HDL-cholesterol).

Estimation of protein was performed using Lowry *et al*, 1951 method and urea using Fawcett and Scott., 1960 method. Estimation of creatinine was conducted using Diagnostic Kit-Dr. Reddy’s Laboratories, Bachupally, Hyderabad, India. The formula used was: ΔAT/ΔAS*2=mg/dl.

### Statistical Calculations

Student’s t-test and probability level of *p*<0.001 were chosen as the criterion of statistical significance.Values reported are mean ± SD. All statistical analysis was performed using One Way ANOVA and expressed as ±SD using SPSS 14.0 Version.

## RESULT AND DISCUSSION

The aim of the present study was to test the effect of the *Elephantopus scaber* L. hexane extract fractions on serum cholesterol, triglyceride concentrations and renal function. It has been previously reported that the methanol and aqueous extracts of *Elephantopus scaber* was reported to decrease the serum lipids and improve serum HDL-cholesterol levels (6).

The abnormally high concentration of serum lipids in the diabetic subjects is due, mainly, to increase in the mobilization of free fatty acids from the peripheral depots, since insulin inhibits the hormone sensitive lipase. Insulin deficiency or insulin resistance may be responsible for dyslipidemia, because insulin has an inhibitory action on HMG-CoA reductase, a key rate limiting enzyme responsible for the metabolism of cholesterol-rich LDL particles. Acute insulin deficiency initially causes an increase in free fatty acid mobilization from adipose tissue. This results in increased production of cholesterol-rich LDL paricles (3). On the other hand, glucagon, catecholamines and other hormones enhance lipolysis. The marked hyperlipidemia that characterizes the diabetic state may, therefore, be regarded as a consequence of the stimulatory action of lipolytic hormones in the fats depots (9). Lowering of serum lipid concentration through reduced dietary intake or drug therapy seems to be associated with a decrease in the risk of vascular diseases (18).

Table 1 shows the effect of fractions of hexane extract ESR on Total Cholesterol, triglycerides, HDL, LDL and VLDL in STZ induced diabetic rats. A considerable decrease in the total cholesterol, triglycerides, VLDL, LDL and an increase in HDL was observed in the groups treated with ES extracts. Among the six fractions, the fourth and the sixth fraction had a better hypolipidemic potential compared to the others. Table 2 shows the effect of fractions of hexane ESR extract on Protein, Urea, Uric acid and Creatinine. After the administration of the ESR, there was an increase in protein and a decrease in urea, uric acid and creatinine.

**Table 1:** Hypolipidemic potential of the extracts of ESR hexane fractions on STZ-induced diabetic rats

Parameter	CHOL (mg/dL)	TRGLY (mg/dL)	HDL (mg/dL)	LDL (mg/dL)	VLDL (mg/dL)

Normal	89.34 ± 3.14	66.27 ± 2.44	59.34 ± 5.29	78.2 ± 2.25	21.6 ± 4.21
Diabetic	201.6 ± 2.2	149.26 ± 3.26	14.68 ± 4.25	144.26 ± 2.24	47.6 ± 3.22
Fraction1	205.4 ± 5.2	76.82 ± 5.62	45.21 ± 4.32	89.48 ± 3.2	25.22 ± 20.17
Fraction2	98.4 ± 5.2	73.81 ± 5.62	54.11 ± 4.32	86.48 ± 3.2	22.7 ± 20.17
Fraction3	97.4 ± 5.2	130.51 ± 5.62	26.00 ± 4.32	134.8 ± 3.2	33.7 ± 20.17
Fraction4	205.4 ± 5.2	75.02 ± 5.62	45.25 ± 4.32	89.48 ± 3.02	35.23 ± 2.17
Fraction5	99.4 ± 5.23	70.81 ± 5.62	53.13 ± 4.32	87.48 ± 3.27	32.7 ± 20.17
Fraction6	92.4 ± 5.2	135.22 ± 5.62	24.23 ± 4.32	13089.8 ± 3.2	25.36 ± 2.17
Insulin treated diabetic rat	92.3 ± 2.23	68.2 ± 2.12	59.00 ± 3.65	79.23 ± 2.21	20.62 ± 2.05

Data represented as mean ± SD values of 6 animals each. Significant values when compared with diabetic control *p*<0.001(ANOVA).

**Table 2:** Renal function tests of the extracts of ESR hexane fractions on STZ-induced diabetic rats

Parameters	Protein (mg/dL)	Urea (mg/dL)	Creatinine (mg/dL)

Normal	8.63 ± 0.58	21.90 ± 2.27	1.02 ± 0.11
Diabetic	3.36 ± 0.58	36.77 ± 2.67	2.10 ± 0.14
Fraction1	8.53 ± 0.37^a^	22.18 ± 2.28	1.22 ± 0.26^a^
Fraction2	7.83 ± 0.04	24.33 ± 2.90	1.32 ± 0.29
Fraction3	5.18 ± 0.09	30.83 ± 3.0	1.70 ± 0.30
Fraction4	5.16 ± 0.02	29.6 ± 2.65	1.75 ± 2.02
Fraction5	6.23 ± 2.56	28.45 ± 3.21	1.62 ± 3.32
Fraction6	7.56 ± 2.32	23.65 ± 1.23	1.40 ± 0.41
Insulin treated diabetic rat	8.44 ± 0.76	21.76 ± 2.04	1.10 ± 0.15

Values are mean±SD of six rats.

a *P*<0.001 significant increase.

Although the exact cause of premature atherosclerosis in diabetes is not well understood, several independent risk factors such as hypertriglyceridemia and hypertension may contribute to coronary heart disease (8). The most common lipid abnormalities in diabetes are hypertriglyceridemia and hypercholesterolemia (20). Hypertriglyceridemia is also associated with metabolic consequences of hypoinsulinemia, insulin resistance and glucose intolerance (29). The development of hypertriglyceridemia in uncontrolled diabetes is a consequence of a number of metabolic abnormalities that occur sequentially. Acute insulin deficiency initially causes an increase in free fatty acid mobilization from adipose tissue, resulting in increased production of VLDL-triglyceride in the liver (3). With longer insulin deficiency, the liver converts free fatty acids into ketone bodies (4). At the same time, lipoprotein lipase activity decreases resulting in impaired clearance of VLDL and chylomicrons from blood (2). VLDL, which is a major carrier of plasma triglycerides in blood, becomes rich in cholesterol and acts as a carrier of cholesterol (13). Significant lowering of total cholesterol and rise in HDL-Cholesterol is a very desirable biochemical state for prevention of atherosclerosis and ischaemic conditions (12). Several studies show that an increase in HDL-Cholesterol is associated with a decrease in coronary risk and most of the drugs that decrease total cholesterol also decrease LDL-cholesterol (28).

In STZ-induced diabetic rats, the rise in blood glucose is accompanied by an increase in serum cholesterol and triglycerides and decrease in HDL-cholesterol. Methanolic and aqueous extracts of *Termanalia catappa* fruit increased the serum HDL-cholesterol in alloxan-induced diabetic rats (15).

Ethanolic extracts of *Hibiscus rosa-sinensis* flower significantly lowered total cholesterol and increased HDL-cholestrol in diabetic rats (19). Also the methanol and aqueous extracts of *Elephantopus scaber* was reported to decrease the serum lipids and improve serum HDL-cholesterol levels (6). In the present study, the effect of ESR and ESL were analysed for their hypolidemic and renal functionalities. According to Table 1 and 2, it is understood that on a long term basis the diabetic state was reverted resulting in restoration to near normal in comparison to the insulin treated groups.

This hypolipidemic effect may be due to an increase in insulin secretion that ultimately led to a decrease in the synthesis of cholesterol and fatty acid. The mechanism of action of the plant extracts appears to be through an increase in insulin level, which increased the activity of lipoprotein lipase and decreased fatty acid synthesis. In the present study, HDL-cholesterol was decreased in diabetic rats. However, administration of the plant extracts brought about a significant increase in HDL-cholesterol. Different extracts of *Elephantopus scaber* brought a greater increase in HDL-cholesterol level in STZ-induced diabetic rats. The plant extracts not only reduced the lipids, but also corrected the metabolic disorders due to diabetes.

Renal diseases is one of the most common and severe complications of diabetes (21). Indeed, a relationship between treatment –related alterations in urea concentration and histopathology of the kidney has been reported in rats (16).

Creatinine, a marker of renal function (24) is significantly increased in diabetic control animals (16). The total protein was restored to the normal range in *Vinca rosea* flower and leaf treated diabetic rats (7). Different extracts of *Elephantopus scaber* brought about a significant increase in serum protein, and a decrease in urea and creatinine levels of STZ-induced diabetic rats. Decrease in protein and increase in serum urea and creatinine concentrations, which is considered as a marker of kidney dysfunction, has been rectified by administration of these extracts in STZ-induced diabetic rats.

## CONCLUSION

We conclude that, the fourth and the sixth fractions derived from hexane extract show a better hypolipidemic potential and a similar trend in renal functions stating the efficiency of *Elephantopus scaber* Linn. as an efficient source of reducing coronary risk and renal failures besides its ability to reduce blood glucose levels. Hence the hexane extract will be subjected to further analysis to derive the compounds responsible for the beneficial activities. The serum cholesterol and triglycerides decreased, whereas HDL-cholesterol increased. Serum protein increased whereas serum urea and creatinine decreased. Hexane extract brought about a better antihyperglycemic effect.
